# The Book World of Medicine and Science

**Published:** 1903-06-06

**Authors:** 


### THE BOOK WORLD OF MEDICINE AND SCIENCE.

THE STORY OF ALCHEMY AND THE BEGINNINGS OF CHEMISTRY. By M. M. PATTISON MUIR, M.A., Fellow and Prælector in Chemistry of Gonville and Caius College, Cambridge. With 17 illustrations. (London: George Newnes, Limited. 1902. Price 1s.)

MR. MUIR'S little volume on alchemy deals with a subject which will always interest the human race, and he treats it in an interesting fashion. To the men to whom we owe the beginnings of modern chemistry he shows full justice; but perhaps he is less than just to the alchemists proper and their search for the philosopher's stone. They made, blindly and with many stumbles, the first step which proverbially costs so much more than those that follow, and no more deserve to be judged according to our standards of scientific method than would Columbus deserve to be despised because he thought when he came to the Bahamas and called them the West Indies that he had set foot on the west coast of Hindostan. Contrasting the modern chemist with his ancestor in the science, Mr. Muir says: "I think it is not unfair to say that the chemist experiments in order that he may liken his imaginings to the facts which he observes"; the alchemist toiled that he might liken the facts which he observed to his imaginings. The difference may be put in another way by saying: The chemist's object is to discover "how changes happen in combinations of the unchanging"; the alchemist's endeavour was to prove the truth of his fundamental assertion, "that every substance contains undeveloped resources and potentialities, and can be brought outward and forward into perfection." There is a good deal to be said for both points of view; and while the former statement represents that of the pure chemist who follows his science for its own sake the latter holds good of much of that application of chemical knowledge to the arts of which we see so much in the present day. It is, moreover, quite conceivable that alchemists when, by some of the lucky chances which occur in all laboratories, they discovered some new substance or produced some new effect, being unable to trace all the steps in the process, themselves believed that they had accomplished some magic feat, and gained an occult control over the powers of nature, when in truth they were only better scientists than they knew. If a man had, for example, drawn out of a stone a liquid which could be changed into a form of air, and then into fire—in other words had made gas from coal—he might be excused if he evolved from the facts a theory that all elements were contained potentially in each one of them. It is easy to see to what wild experimenting such a theory might lead, and also, in an age when scholastic speculation was rife, to what still wilder philosophising on the causes of things. The shores of the ocean of science are strewn with the broken fragments of discarded theories, many of them not more wise than those of the alchemists, and of much more recent date. This of itself may explain much of that vagueness of statement which Mr. Muir quotes with condemnation from the writings of the alchemists. He also takes no account of a theory regarding the alchemical writings which is held by a considerable number of people nowadays, namely, that the apparently cryptic sayings of the alchemists had a spiritual rather than a scientific meaning, and conveyed to the initiated words of moral counsel and encouragement. The esoteric form was very necessary when the Church doomed to the fire and the rack all who differed in the smallest degree from the received theology. Thus interpreted, Paracelsus' saying: "Destruction perfects that which is good; for the good cannot appear on account of that which conceals it;" or that of Michael Sendivogius: "The bodily nature of things is a concealing outward vesture," have a perfectly comprehensible meaning, though they do not in any way express a chemical formula. Mr. Muir's little book, with its curious lore and quaint illustrations, should send many readers to study the things accomplished by these long-forgotten students.

THE MEDICAL TREATMENT OF GALL-STONES. By J. H. KEAY, M.A., M.D. (London: Rebman, Limited. 1902. Pp. 126. 3s. net.)

DR. KEAY, several years ago, suffered from gall-stones. He consulted an eminent specialist and ultimately consented to submit himself to operation. One morning, just before the proposed operation, Dr. Keay passed half a dozen gall-stones; he gradually then regained his health and strength, and happily has now the vigour to write this little work suggesting that if "medical and hygienic means adapted to the requirements of each individual be resorted to early enough in the course of the disease, in very few cases indeed need the question ever arise as to whether the patient should submit to the brilliant, yet often dangerous and even fatal, remedies of the gall-stone surgeon." Of course, in a sense, Dr. Keay is right; but what are "the medical and hygienic means adapted to the requirements of each individual" that Dr. Keay so firmly believes in? Simply, we gather, those which are employed by everyone and which so frequently fail. What practitioner does not attempt, when he meets with a case of "gall-stones," to remove "errors of digestion, worry, constriction by dress," and who does not order mineral waters and narcotics, prescribe drugs "that promote flow of healthy bile," and give turpentine, ether, and olive oil in turn? Is it not contrary to the general experience of the profession to say that "the records of gall-bladder surgery during the past ten years have already shown that the anticipated results have not been realised?" We cannot think that Dr. Keay, despite the happy result of his own illness, will find many to believe with him, that "unless in the most exceptional cases, the gall-bladder sufferer will derive more real and lasting benefit from hygienic and medical treatment than operation."

